# Vibrio fluvialis Bacteremia With Bullous Cellulitis

**DOI:** 10.7759/cureus.94545

**Published:** 2025-10-14

**Authors:** Arko S Dhar, Arif Zulfiqar

**Affiliations:** 1 Internal Medicine, University of Mississippi Medical Center, Jackson, USA; 2 Family Medicine, University of Mississippi Medical Center, Jackson, USA

**Keywords:** bacteremia, cellulitis, infectious diseases, vibrio fluvialis, vibriosis

## Abstract

*Vibrio fluvialis* is a gram-negative bacillus commonly implicated in outbreaks of gastroenteritis. The bacterium has been identified as an emerging pathogen in recent years, with increasing reports of extraintestinal manifestations. This report discusses a case of bacteremia presumed secondary to bullous cellulitis of the left lower extremity, with blood cultures positive for *Vibrio fluvialis *and *Streptococcus dysgalactiae*. The patient presented with progressively worsening quadriplegia and falls, with one week of progressive discoloration of the left lower extremity. He denied any gastrointestinal symptoms and had no recent history of seawater exposure or seafood consumption. Initial inpatient management consisted of parenteral antibiotics, although complications eventually necessitated an above-the-knee amputation (AKA).

## Introduction

*Vibrio fluvialis* is a halophilic gram-negative bacterium commonly present in seawater, brackish water (i.e., lagoons and estuaries), and freshwater ecosystems [1,2]. Major routes of transmission include exposure to contaminated water, as well as ingestion of contaminated seafood, such as raw oyster and shellfish [1,2]. Comparable to other *Vibrio* species, the pathogen has largely been implicated in outbreaks of gastroenteritis. Numerous extraintestinal manifestations have been reported, however, including necrotizing fasciitis, hepatic abscess, cholangitis, pneumonia, peritonitis, and urinary tract infection [3-8]. Recent Centers for Disease Control and Prevention (CDC) statistics reveal that *Vibrio fluvialis* has been the causative agent in approximately 6.9% of confirmed annual United States (US) vibriosis cases, with the total case count being the fifth highest among all *Vibrio* species [9]. 

*Vibrio fluvialis* has been classified as an emerging pathogen in recent years, and its public health relevance is only likely to grow [1]. A warming climate, leading to warmer aquatic environments, has already led to *Vibrio* outbreaks occurring at higher latitudes, including US regions as far north as the mid-Atlantic and New England [10]. Natural disasters, such as flooding precipitated by hurricanes, have also led to greater exposure to *Vibrio *species [10]. Here, we report a case of bacteremia secondary to left lower extremity cellulitis, with blood cultures taken on day three of admission growing *Vibrio fluvialis*. The patient ultimately developed diffuse myonecrosis of the left lower extremity, necessitating above-the-knee amputation (AKA). This case adds to the growing body of literature involving extraintestinal manifestations of *Vibrio fluvialis *infection. 

## Case presentation

A 61-year-old African-American male patient with a past medical history of type 2 diabetes, peripheral neuropathy, hypertension, and chronic lymphedema presented to the emergency department due to an unwitnessed fall at home. On initial emergency medical services evaluation, the patient was noted to have globalized weakness and quadriplegia. He denied any loss of consciousness but endorsed a history of recent falls, along with chronic right upper extremity weakness. The patient also reported developing dark discoloration, swelling, and tenderness in his left lower extremity beginning one week prior, with blistering and “dark-reddish” fluid drainage. The patient lived in a coastal state and worked at a restaurant, primarily involved in handling chicken. Seafood was noted to be served at the restaurant. The patient’s home medications included gabapentin, amlodipine, furosemide, and hydrochlorothiazide. He had no pertinent prior surgical history. The patient denied any smoking history and use of illicit drugs but did endorse a history of alcohol consumption (one standard drink per week).

The patient’s initial vital signs and notable lab findings upon presentation to the emergency department are shown in Tables 1-2, respectively. No prior laboratory results were available for comparison. On physical examination, the patient was alert but had left hemiparesis and impaired sensation. He had diminished right upper extremity motor function against gravity. Bilateral lower extremity edema was appreciated, with significant scaling, skin abrasions, and hemorrhagic bullae, comparatively worse on the left lower extremity. 

**Table 1:** Initial vital signs upon presentation

Vital sign	Measured value	Reference range
Temperature (°C)	36.4	36.1-37.2
Blood pressure (mmHg)	163/98	90-130/60-80
Heart rate (beats/minute)	62	60-100
Oxygen saturation (%)	98	≥95
Respiratory rate (breaths/minute)	18	12-20

**Table 2:** Notable initial laboratory workup

Test	Measured value	Reference range
White blood cell count (K/​​mm^3^)	15.4	4-10
C-reactive protein (mg/L)	117.2	<0.2-10
Erythrocyte sedimentation rate (mm/hr)	83	12-14
Sodium (mmol/L)	132	136-145
Hemoglobin (g/dL)	11.3	13-17
Hematocrit (%)	35.4	40-50
Aspartate aminotransferase (U/L)	130	0-40
Alanine aminotransferase (U/L)	52	0-41

**Figure 1:**
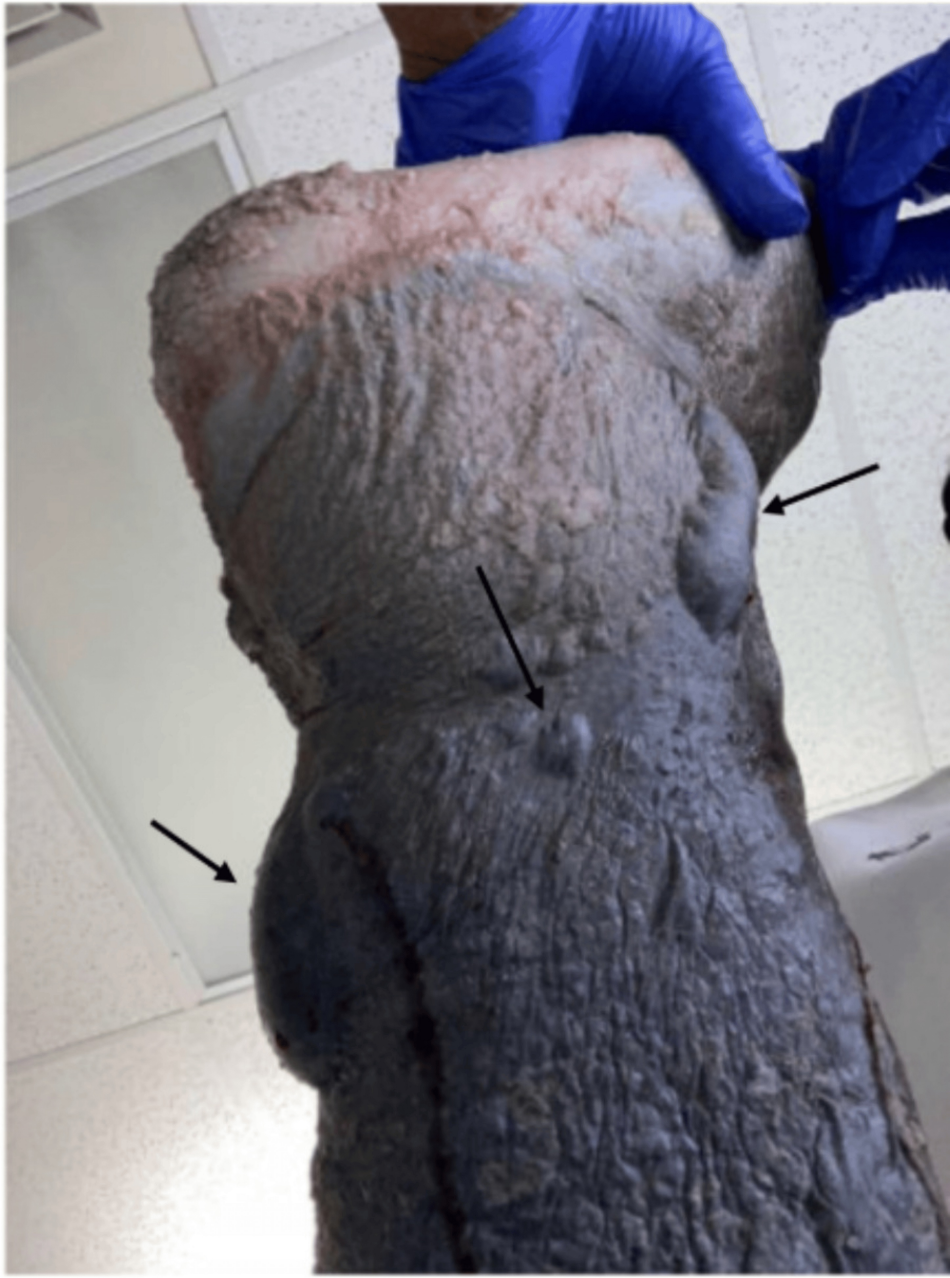
Left lower extremity cellulitis with hemorrhagic bullae on day 1 of hospitalization. Bullae are indicated by black arrows.

The patient was administered intravenous (IV) dexamethasone in the emergency department, given computed tomography (CT) findings of a large posterior disc herniation between the third and fourth cervical vertebrae (C3-C4), causing cord compression and severe central stenosis. CT imaging of the head was unremarkable. The wound care nursing team was consulted for management of bullous left lower extremity cellulitis in the setting of chronic lymphedema, as shown in Figure 1. He was admitted thereafter for neurosurgical evaluation for possible surgical intervention.

Initial cultures taken on the first day of admission grew *Streptococcus dysgalactiae* in one of two sets, with the patient’s left lower extremity cellulitis presumed to be the source. A urine culture speciated for pan-susceptible *Enterococcus*. The patient was started on IV vancomycin (renally dosed), and the infectious diseases team was consulted. Coverage was later broadened by adding doxycycline along with IV cefepime (renally dosed). Repeat blood cultures taken on the third day of admission grew gram-positive cocci in chains, along with gram-negative rods, in two out of two sets. The gram-negative rods later speciated for *Vibrio fluvialis*, which was found to be pan-susceptible. The patient’s wound culture swab at this point grew *Enterobacter cloacae* complex (multidrug-resistant) and *Streptococcus dysgalactiae. *Antibiotic coverage with cefepime was transitioned to meropenem, with continuation of doxycycline. Sensitivity results for *Vibrio fluvialis* toward different antibiotics are shown in Table 3. 

**Table 3:** Sensitivity results for Vibrio fluvialis toward different antibiotics

Antibiotic	Susceptibility
Amikacin	Sensitive
Ampicillin/sulbactam	Sensitive
Cefepime	Sensitive
Cefotaxime	Sensitive
Ciprofloxacin	Sensitive
Gentamicin	Sensitive
Levofloxacin	Sensitive
Piperacillin/tazobactam	Sensitive
Trimethoprim/sulfa	Sensitive

**Figure 2:**
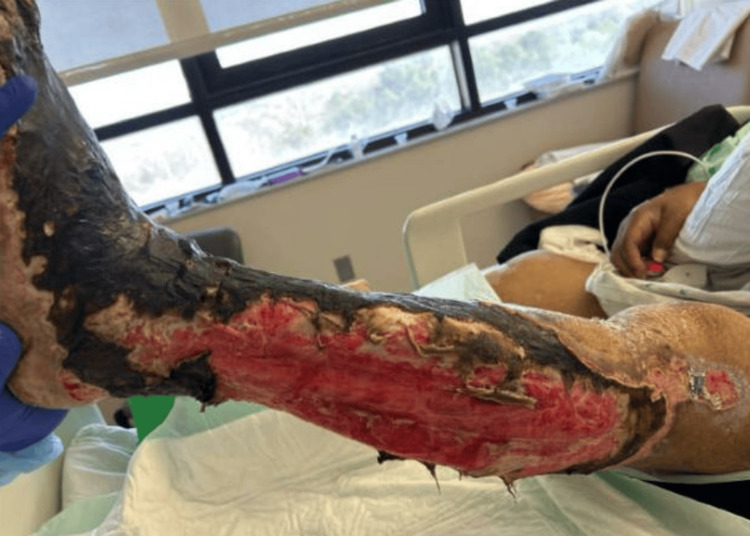
Left lower extremity with extensive skin necrosis on day 47 of hospitalization.

Additionally, around this time, the patient’s clinical status worsened, with concerns for polymicrobial sepsis. The patient had developed multiple electrolyte abnormalities in the setting of rhabdomyolysis-induced acute kidney injury, requiring hemodialysis. Regular wound care was continued for his left lower extremity wounds, which appeared to worsen (Figure 2). CT imaging of the left lower extremity correlated with clinical findings, revealing superficial soft tissue edema and swelling, with diffuse involvement of the anterior and posterior compartments. No gas production was noted. The plastic surgery team was consulted for possible source control, but they recommended continued wound care. The patient was deemed medically unstable for surgical intervention, both from the standpoint of plastic surgery and neurosurgery.

Later during the hospital stay, the patient developed septic shock, necessitating transition to the intensive care unit (ICU). The ICU course was complicated by acute hypoxemic respiratory failure requiring tracheostomy, along with small bowel obstruction, managed conservatively. The patient was continued on meropenem, with the addition of vancomycin and fluconazole for broader antimicrobial coverage. The patient’s hemodynamic status eventually improved in the ICU, and the vascular surgery team was consulted for possible left lower extremity amputation, given the development of extensive necrosis (Figure 2). 

The patient proceeded to undergo irrigation and debridement, followed by AKA of the left lower extremity. Diffuse myonecrosis was noted intraoperatively. 

The patient was eventually discharged to a long-term rehabilitation facility but presented to the emergency department three days later due to sudden-onset hypotension and altered mental status. Days following readmission, he succumbed to his extensive comorbidities.

## Discussion

We present a complicated infectious case, with *Vibrio fluvialis* being one of multiple organisms contributing to lower extremity bullous cellulitis and eventual sepsis. No gastrointestinal symptoms, such as nausea, vomiting, or diarrhea, had been reported on admission. While *Vibrio fluvialis* classically presents as a self-limited gastroenteritis, complicated cases, including many extraintestinal presentations, have been reported. The majority of bacteremia cases involving *Vibrio fluvialis* have occurred in the setting of predisposing conditions or a definitive history of seafood exposure. Lai et al. reported the first case of *Vibrio fluvialis* bacteremia manifesting in a patient with cirrhosis, a known risk factor for *Vibrio* infection [11]. The patient in the report of Lai et al. suffered from gastrointestinal complications of *Vibrio* infection, namely severe watery diarrhea [11].

Multiple presentations of *Vibrio fluvialis* bacteremia secondary to hepatobiliary causes have been reported. Kitaura et al. reported a case of *Vibrio fluvialis* bacteremia in Japan in the setting of a liver abscess [4]. The patient in this report had a recent dietary history of sashimi consumption, which was presumed to be the cause of his infection [4]. Itoh et al. reported a similar case of *Vibrio fluvialis* bacteremia in Japan in a patient with a dietary history of frequent supermarket sushi consumption [5]. Both patients were successfully treated with inpatient parenteral antibiotics [4,5]. Lazzari et al. reported a fatal case of *Vibrio fluvialis* cholangitis in Europe, which was complicated by hepatic abscess and bacteremia [12]. The patient in the report of Lazzari et al. neither presented with any gastrointestinal symptoms nor had any history of seafood exposure - of note, however, the patient was an 85-year-old male individual with multiple comorbidities and chronic steroid use [12].

Aside from bacteremia, cases of dermatologic pathologies secondary to *Vibrio fluvialis* have been reported. Smith III et al. reported a case of hemorrhagic bullae in a patient diagnosed with *Vibrio fluvialis* bacteremia [13]. The patient in this case, however, endorsed a recent dietary history of raw seafood consumption [13]. He was reported to have profuse diarrhea preceding admission and was found to have fecal matter running over his legs while bed-bound, presumably worsening his wound infection [13]. Huang and Hsu documented a case of a patient who suffered from hemorrhagic cellulitis secondary to *Vibrio fluvialis* infection, with cerebritis emerging as a superimposed complication [14]. The patient in question had suffered multiple fire ant stings, with subsequent exposure to brackish water [14]. The patient we report likewise suffered from cellulitis, but his neurologic deficits on presentation were found to be related to cervical spine herniation as opposed to an infectious process. 

In our case, the patient lacked any significant history that would predispose to an immunocompromising state. Of note, initial CT imaging did reveal diffuse reduced bone marrow signal, suggestive of possible marrow hyperplasia with an underlying hematologic pathology. Bone marrow biopsy was never pursued, however. From the standpoint of infectious exposures, the patient did not endorse any direct exposure to or consumption of seafood preceding his admission. His work primarily involved marinating chicken at a restaurant, although this place of work was known to serve seafood dishes as well. Of note, the patient did live in a coastal state. While inconclusive, the aforementioned reasons are all factors to consider in understanding how this patient could have developed *Vibrio fluvialis* bacteremia. 

## Conclusions

Given the increasing prevalence of* Vibrio fluvialis* and the high variability in presentation, more investigation involving the pathogen is warranted. This case report highlights a relatively complicated infectious case of polymicrobial sepsis presumed secondary to left lower extremity bullous cellulitis. *Vibrio fluvialis*, one of the organisms grown on initial blood cultures, likely played a major role in the progression of the patient’s lower extremity wounds, which eventually required AKA for source control. The case highlights an extraintestinal manifestation of vibriosis, which is a rarer, albeit frequently documented, finding in the literature. The rapid worsening of the patient’s lower extremity pathology underscores the need for vigilance when managing this pathogen, for which there remains no widely established treatment guideline.
